# Large-scale and significant expression from pseudogenes in *Sodalis glossinidius* – a facultative bacterial endosymbiont

**DOI:** 10.1099/mgen.0.000285

**Published:** 2020-01-10

**Authors:** Ian Goodhead, Frances Blow, Philip Brownridge, Margaret Hughes, John Kenny, Ritesh Krishna, Lynn McLean, Pisut Pongchaikul, Rob Beynon, Alistair C. Darby

**Affiliations:** ^1^​ Institute of Integrative Biology, University of Liverpool, Crown Street, Liverpool, L69 7ZB, UK; ^2^​ School of Science, Engineering and Environment, Peel Building, University of Salford, M5 4WT, UK; ^3^​ Department of Entomology, Cornell University, Ithaca 14853, NY, USA; ^4^​ Centre for Genomic Research, Institute of Integrative Biology, University of Liverpool, Crown Street, Liverpool, L69 7ZB, UK; ^5^​ IBM Research UK, STFC Daresbury Laboratory, Warrington, WA4 4AD, UK

**Keywords:** pseudogenes, *Sodalis glossinidius*, endosymbiont, transcriptome

## Abstract

The majority of bacterial genomes have high coding efficiencies, but there are some genomes of intracellular bacteria that have low gene density. The genome of the endosymbiont *Sodalis glossinidius* contains almost 50 % pseudogenes containing mutations that putatively silence them at the genomic level. We have applied multiple ‘omic’ strategies, combining Illumina and Pacific Biosciences Single-Molecule Real-Time DNA sequencing and annotation, stranded RNA sequencing and proteome analysis to better understand the transcriptional and translational landscape of *Sodalis* pseudogenes, and potential mechanisms for their control. Between 53 and 74 % of the *Sodalis* transcriptome remains active in cell-free culture. The mean sense transcription from coding domain sequences (CDSs) is four times greater than that from pseudogenes. Comparative genomic analysis of six Illumina-sequenced *Sodalis* isolates from different host *Glossina* species shows pseudogenes make up ~40 % of the 2729 genes in the core genome, suggesting that they are stable and/or that *Sodalis* is a recent introduction across the genus *Glossina* as a facultative symbiont. These data shed further light on the importance of transcriptional and translational control in deciphering host–microbe interactions. The combination of genomics, transcriptomics and proteomics gives a multidimensional perspective for studying prokaryotic genomes with a view to elucidating evolutionary adaptation to novel environmental niches.

## Data Summary

The Pacific Biosciences assembly and annotation have been submitted to the European Nucleotide Archive under accession nos LN854557–LN854560. Illumina sequence reads for the six additional *Sodalis* isolates are available under project accession PRJEB9474 (accession nos ERR2036891–ERR2036896). RNAseq data are available under project PRJEB20150. The mass spectrometry proteomics data have been deposited to the ProteomeXchange Consortium via the PRIDE [1] partner repository with the dataset identifier PXD007068.

**Impact Statement**

Bacterial genes are generally 1 kb in length and organized efficiently (i.e. with few gaps between genes or operons), and few open reading frames (ORFs) lack any predicted function. Intracellular bacteria have been removed from extracellular selection pressures acting on pathways of declining importance to fitness, and thus these bacteria tend to delete redundant genes in favour of smaller functional repertoires. In the genomes of endosymbionts with a recent evolutionary relationship with their host, however, this process of genome reduction is not complete; genes and pathways may be at an intermediate stage, undergoing mutation linked to reduced selection and small population numbers being vertically transmitted from mother to offspring in their hosts, resulting in an increase in the abundance of pseudogenes and reduced coding capacities. A greater knowledge of the genomic architecture of persistent pseudogenes, with respect to their DNA structure, mRNA transcription and even putative translation to protein products, will lead to a better understanding of the evolutionary trajectory of endosymbiont genomes, many of which have important roles in arthropod ecology.

## Introduction

The genomes of intracellular parasites and endosymbiotic bacteria evolve under conditions that are fundamentally different from those of free-living organisms [2]. In many arthropod systems, bacteria can provide nutrients that are otherwise scarce to their host (such as B vitamins absent from blood meals, or essential amino acids absent from plant sap), in exchange for host provision of protection, nutrition and mechanisms for vertical or horizontal transmission [3, 4]. Obligate intracellular symbionts are maintained by the host and have evolved strategies that ensure their vertical transmission to the next generation of hosts. Ultimately, this intracellular lifestyle, small population size and strict vertical transmission can result in extremely reduced genomes [2, 4–7]. The general theory and process of this extreme genome reduction has been well studied using genomic data for intracellular bacteria, including endosymbionts such as *Buchnera* in aphids [8, 9] and *Wigglesworthia* in tsetse flies [10]. However, gene loss is not limited to obligate intracellular pathogen/symbionts with strict vertical transmission, it is also observed in free-living bacteria and facultative symbionts [11].

One of the most important mechanisms for gene loss is that of pseudogenization, resulting from the accumulation of nonsense mutations in protein coding sequences [2]. These mutations putatively silence the gene at the genomic level, resulting in theoretically non-functional genes/proteins [12]. Prokaryotic pseudogenes generally exist at levels between approximately 1 and 5 % [13]. Comparative genomic analysis between closely related strains suggests that pseudogenes are often associated with reduced selective pressure on redundant gene sets, allowing mutation to accumulate and inactivate genes. This has been observed as *Salmonella* changes host range or utilizes a new environment [14]. The low level of pseudogenes in most bacteria suggests that they are removed rapidly from the genomes due to strong selection for genome efficiency [12]. There are, however, examples among the intracellular pathogens and endosymbionts of high levels of pseudogene presence, reducing coding capacity down towards 50 % in *Sodalis glossinidius* [15] and *Mycobacterium leprae* [16]. Likewise, pseudogenes can persist for long periods – the mean half-life of *Buchnera aphidocola* pseudogenes has been estimated to be 24 million years [17]. Pseudogenes have been well studied in the context of comparative genomics to understand how gene loss has shaped bacterial genomes [18], but whether they continue to contribute to the genetic capabilities of the bacterium has seldom been assessed [19]. It could, for instance, be suggested that if pseudogene-derived transcription retains some form of *cis*/*trans* regulatory function, then this could select for pseudogene retention in the genome [20]. It is also clear that under some circumstances, specifically where polymerase infidelity corrects for a frameshift within homopolymeric tracts at the transcriptional level, pseudogenes can still produce functional proteins that contribute to the fitness of the bacterium [21].

In this study we aim to understand the importance of pseudogenes in bacterial genome evolution in a model of a degrading bacterial genome, that of *S. glossinidius. Sodalis* is a facultative intracellular secondary endosymbiont of the tsetse fly (Diptera: *Glossina*). The variable frequency of *Sodalis* in natural populations suggests that *Sodalis* is not an obligatory component of the tsetse microbiome [22], however, the occurrence of *Sodalis* in natural populations has been linked to an increased capacity of tsetse to vector African trypanosomes [23]. Interestingly, *Sodalis* has a relatively large genome for a facultatively intracellular endosymbiont (~4 Mbp) and two genome annotations suggest that pseudogene levels are between 29 % [15] and 38 % [24] of the total gene content. The *S. glossinidius* genome has amongst its coding repertoire, systems for flagella, transmembrane transport [15], quorum sensing [25] and, of note, type III secretion systems, encoded by three *Sodalis* symbiosis regions (SSR 1–3), which are analagous to pathogenicity islands [e.g. *Salmonella* pathogenicity islands (SPIs) [26]] and have been implicated in establishing or maintaining symbiosis [27]. By combining the latest high-throughput sequencing and proteomics methods, we hope to shed light on potential post-transcriptional regulatory mechanisms that may be mitigating any potential deleterious effects. At the RNA level, riboswitches [28] or small RNAs (sRNAs) – short, 50–300 bp transcripts mediated by imperfect base pairing interactions – have been shown to regulate genes in this manner [29]. DNA methylation could also serve as a mechanism by which to control transcription and/or translation [30, 31].

*S. glossinidius* represents an ideal system in which to test hypotheses surrounding pseudogene functionality and their evolution, as the organism maintains an unusually reduced coding capacity, yet remains amenable to cell culture, allowing for sufficient DNA, RNA and peptides to be extracted for poly-omic analyses. First, assuming genes with nonsense mutations are non-functional and therefore costly to the cell, pseudogenes should be evolving rapidly and be removed from the genome. Second, if pseudogene transcription or translation is deleterious, pseudogenes should be transcriptionally and translationally silent. Third, given hypothesis two, we can expect there to be genetic mechanisms to silence pseudogenes, and we will be able to identify genetic and transcriptional features that determine pseudogene status using a combination of genomic, expression and proteomic analysis. To that end, we tested these hypotheses by (1) establishing pseudogene content and evolution using pan-genome data; (2) evaluating genome-wide methylation data and negative-strand expression to elucidate potential expression control mechanisms; and (3) correlating mRNA and protein expression levels to understand functional control of pseudogenes.

## Results

### *Sodalis* genomics

To provide an updated, accurate reference for transcriptome mapping of the *S. glossinidius* isolate used in this study, we sequenced *de novo* a *S. glossinidius* (from the host *Glossina morsitans morsitans*) isolate (SgGMMB4) using Pacific Biosciences (PacBio) sequencing. A single SMRTcell produced a total of 48 519 reads, with a mean length of 10 290 bp and mean read score of 0.85. The chromosome was assembled into a single 4.1 Mbp contig and one copy of the three plasmid sequences pSG1 (90 747 bp), pSG2 (38 394 bp) and pSG3 (10 640 bp). Previously published annotations from the GMM4 isolate [15, 24, 32] were used, alongside a manually curated PROKKA-generated annotation of the PacBio-sequenced isolate, to generate a new annotation of our *Sodalis* sequence [33]. The overall mean genome GC content is 54.4 % and the pseudogenes and coding domain sequences (CDSs) have a similar GC of ~55.5 %. The revised annotation presented contains 3336 putative CDSs and 2286 putative pseudogenes. In addition, 43 putative riboswitch domains, belonging to 7 families, were identified (Table S1, available in the online version of this article). A prodigal (v. 2.6.2) [34] gene prediction including a ribosome-binding site (RBS) identification scan suggested a prevalence of standard methionine-coding ATG start codons (78 %), which decreases in the case of pseudogenes (67 %). GTG start codons are the next most common, found in 14 % of all genes, increasing to 21 % of the total number of pseudogenes, followed by TTG (8 % overall; 12 % of pseudogenes). Sixty-one per cent of all genes (48 % of pseudogenes) have an RBS predicted within 5–10 bp of the start codon and 26 % of all genes (38 % pseudogenes) have no discernible RBS. A mummerplot [35] of plasmid pSG1 revealed a 6371 bp tandem repeat encompassing the inactive type IV secretion system operon that is not present in the original *Sodalis* sequencing and annotation experiments, perhaps due to a collapsed repeat missed by first-generation sequencing assembly. Pseudogenes tend to be enriched for activities such as transmembrane transport, including metal ions (GO:0099132; 23 pseudogenes/10 CDSs; Fisher exact test, *P*=0.001); transposition (GO:0006313; 52 pseudogenes/14 CDSs; *P*<0.0001); receptors (GO:0004872; 27 pseudogenes/13 CDSs; *P*<0.0001) and glycerol metabolic processes (GO:0006071; 6 pseudogenes/0 CDSs; *P*<0.01).

### *Sodalis* transcriptomics

**Fig. 1.**
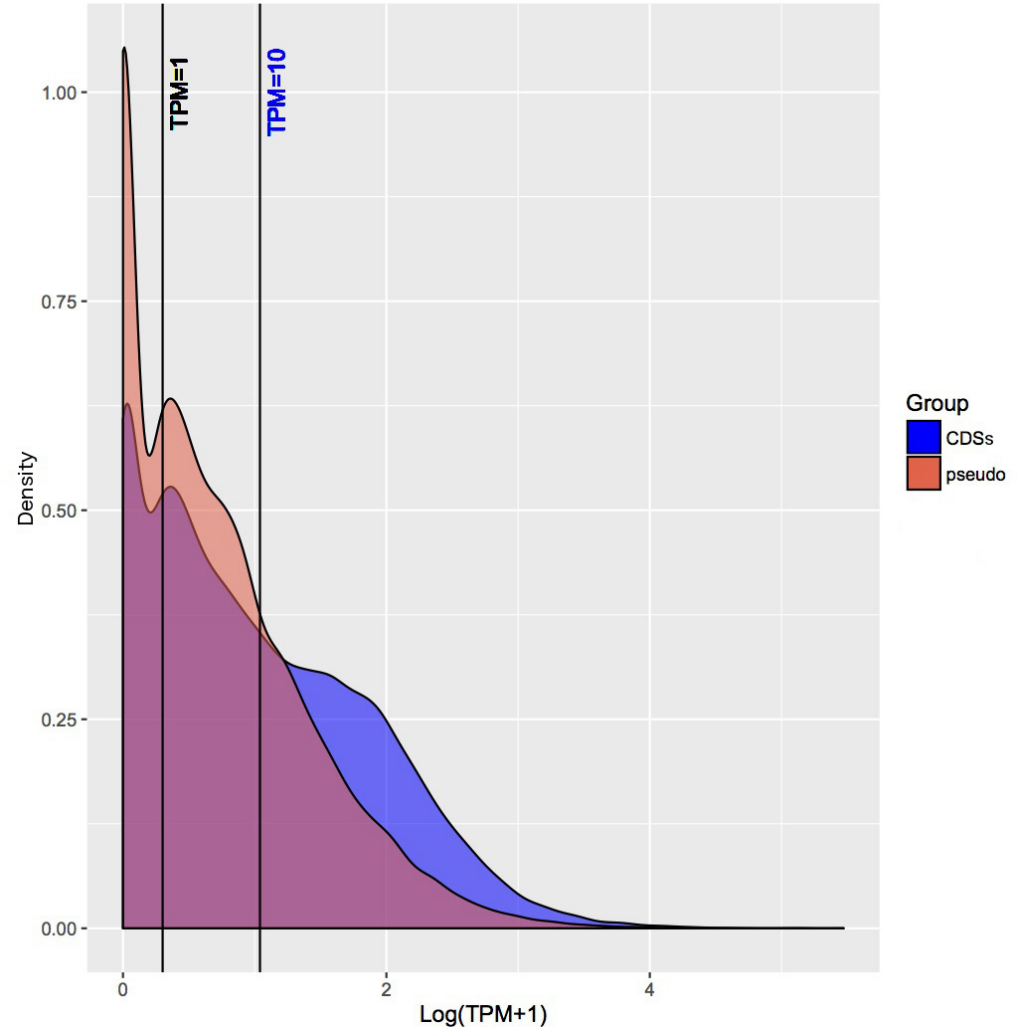
(a) Sense and antisense mean transcription of CDSs (blue) and pseudogenes (red) in three cell-free cultures of *S. glossinidius *: early log phase (ELP), late log phase (LLP) and late stationary phase (LSP). Transcripts per million (TPM) derived from EdgeR counts per million have been transformed to log(TPM+1) to enable the presentation of zero transcription. (b, c) Density plot of expression [log (TPM+1)] showing transcription for all three cell-free culture conditions, grouped by CDS (blue) or pseudogene (red). Lines represent TPM=1 and TPM=10, representing two different minimum thresholds to be considered as activity. Panel (b) is sense transcription only, and panel (c) displays all transcription. The CDSs can be seen to show a greater degree of increased expression levels than the pseudogenes (red). Overlapping low-expression CDSs and pseudogenes highlight the difficulty in identifying pseudogenes using transcription levels.

To ascertain whether pseudogenes are being transcribed, or if their transcription is being regulated throughout growth, stranded RNA sequencing was performed on three replicates in three conditions across a bacterial growth experiment in cell-free media (early log phase, ELP; late log phase, LLP; late stationary phase, LSP). Fig. 1a shows boxplots of mean sense and antisense transcription [log (transcripts per million [TPM]+1)] for each condition. From the density plots of sense transcription (Fig. 1b) and of overall transcription (Fig. 1c), it can be seen that there is a clear signal of no transcription from putatively inactive genes (logTPM+1=0). Other studies have used TPM of ≥1[36] or ≥10 [37] as an indicator of activity, which are displayed on each of these figures. For this study, we have defined putatively active genes as having an arbitrary TPM value ≥1 in all three biological replicates in at least one condition. Genes with TPM≥10 in all three biological replicates in at least one condition are additionally described as being active. Fifty-three per cent of all combined genes and pseudogenes (3087; 2237 CDSs and 850 pseudogenes) exhibited active sense transcription (TPM≥10) in any given condition, with an additional 1191 (547 CDSs and 644 pseudogenes) being putatively active (TPM≥1; a total of 73.7 % of all genes and pseudogenes). Additionally, 1088 genes (703 CDSs and 385 pseudogenes) showed active antisense transcription, with an additional 993 genes (629 CDSs and 364 pseudogenes) exhibiting putatively active antisense transcription according to the same rules.

Across all conditions, mean sense CDS expression (TPM=245.33) was significantly greater than that of pseudogenes (TPM=60.96; Mann–Whitney U test; *W*=46797000, *P*-value<2.2e-16). Mean antisense CDS expression (TPM=24.74) was also significantly greater than that for pseudogenes (TPM=13.64; *W*=37651000, *P*-value<2.2e-16). Actively expressed pseudogenes (TPM ≥10) are enriched for organonitrogen compound metabolic processes (GO:1901564; 51 pseudogenes; 31 CDSs; *P*<0.0001). Putatively expressed pseudogenes (TPM ≥1 and TPM <10) tend to be involved in transposase activity (GO:0006313; 52 pseudogenes; 14 CDSs; *P*<0.0001).

Differential expression analysis suggests that, of the actively transcribed genes, 938 CDS and 219 pseudogenes are being differentially expressed between either LLP or LSP when compared to ELP growth false discovery rate (FDR ≤0.05). In terms of antisense expression: 219 CDSs and 106 pseudogenes showed differential antisense expression between timepoints. Differentially expressed pseudogenes are involved with nucleic acid and organic cyclic compound binding activities (GO:0003677 and GO:0097159).

**Fig. 2.**
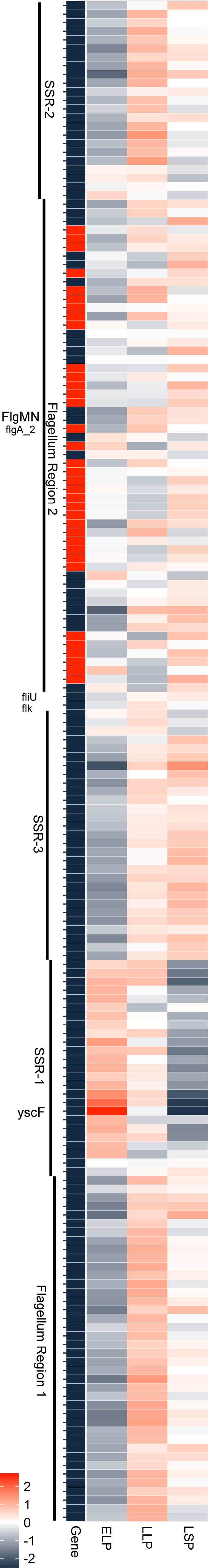
*
S. glossinidius* flagellum and *Sodalis* symbiosis region expression, summarized by general ‘region’ (left bar). Two genes not covered by general region bars are fliU (top) and flk (bottom). CDSs (blue) and pseudogenes (red) are displayed as the first coloured column. Early log phase (ELP), late log phase (LLP) and late stationary phase (LSP) expression is displayed as a heatmap of log fold change relative to average expression, where red signifies upregulation and blue represents downregulation.

Two related examples of specific gene degradation putatively associated with positive or negative selection are the type III secretion and motility systems. *S. glossinidius* has two broad regions that encode for flagella-related proteins – one intact flagellum region (flagellum region 1) and one undergoing significant degradation (flagellum region 2, Fig. 2) – and three T3SS regions (SSR1–3), all largely intact. Flagella-coding genes were significantly more likely to exhibit sense expression (chi-square=31.06; *P*<0.0001) and more likely to be differentially expressed than pseudogenized flagella genes in flagellum region 2 (chi-square=35.27; *P*<0.0001; Fig. 2). The intact flagellum region is upregulated in the late log phase and downregulated in the early log phase growth relative to average expression. SSR-1 is upregulated in the early log phase and downregulated in the late stationary phase. SSRs 2 and 3 are downregulated in the early growth and upregulated in the late log and late stationary phases, respectively (Fig. 2).

**Fig. 3.**
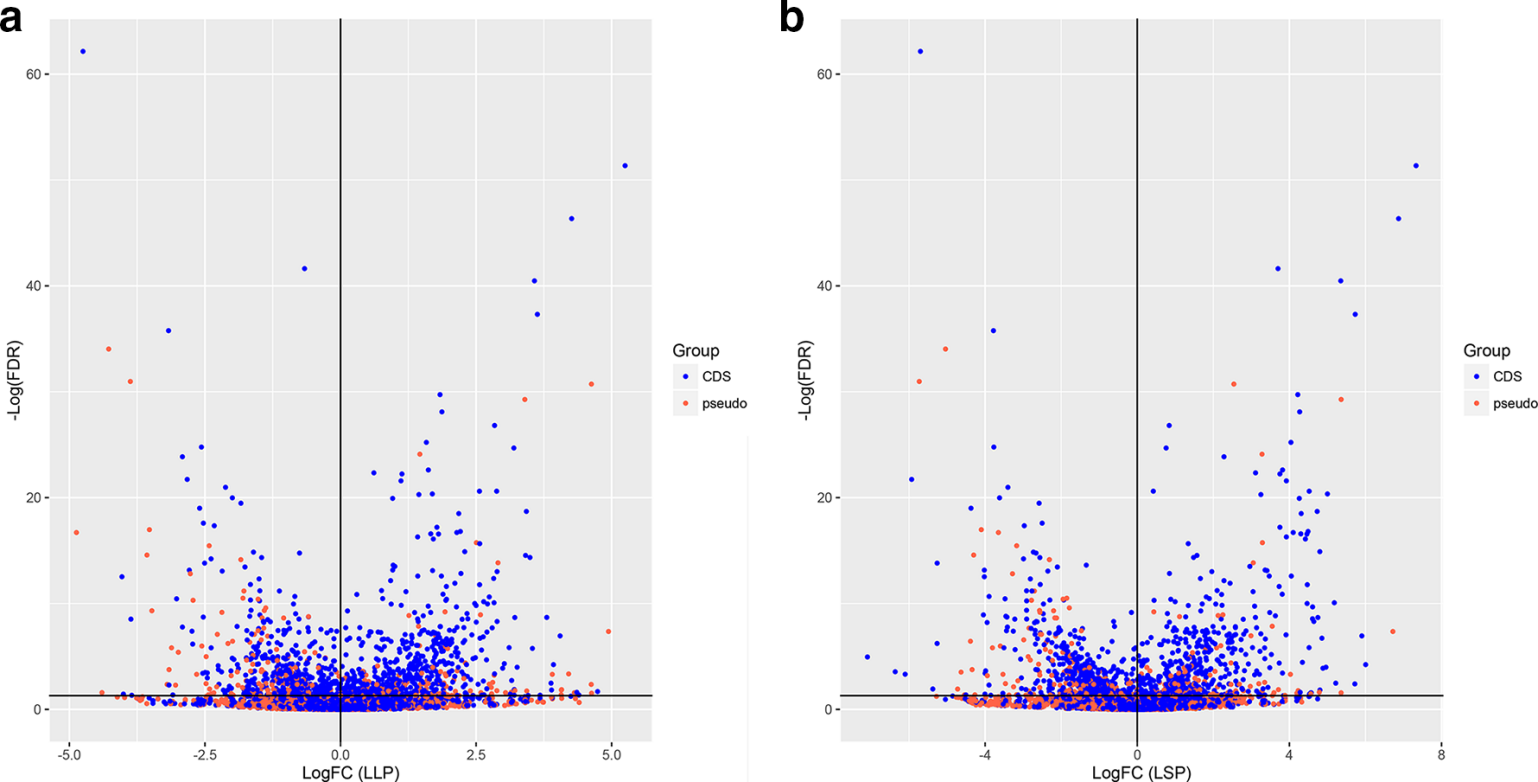
–Log_2_FC differential expression plotted against negative logFDR (false discovery rate), determined from EdgeR. (a) LLP and (b) LSP transcription. Whilst CDSs (blue) represent the majority of differentially expressed genes, some pseudogenes (red) are significantly differentially expressed in either condition.

Fig. 3 displays volcano plots of log fold change against negative log FDR for LLP (Fig. 3a), and for LSP (Fig. 3b), versus ELP. Each shows that some pseudogenes are highly likely to be differentially expressed between conditions.

### SgGMMB4 proteome

To support pseudogene annotation, and to assess whether transcription may relate to translation, we performed proteomic analysis from pooled bacterial cells from all conditions in the cell-free growth experiment. The PROKKA gene models and the six-frame translations comprised a total of 5625 and 6769 proteins, respectively. With our MS/MS search on the combined search database, we identified 1503 peptides attributable to the PROKKA annotation. In two instances, an alternative start codon in the annotation (GTG/Val) was manually changed to ATG to encode a methionine residue, in order to match. We identified 34 pseudogenes from previous annotations that corresponded to the presence of a protein product in our data (Supplementary Data 5 and Table S2). Seven pseudogenization events that split ancestral orthologues into two or more open reading frames (ORFs) were verified by observation of peptides from a protein product of one of the resultant ORFs. A further 27 peptides were detected, corresponding to (single-ORF) pseudogene predictions from the original annotation. These were reclassified as CDSs and are detailed in Table S2. It should be noted that these identified proteins are the *representative proteins* from each protein group, as reported by ProteoAnnotator. ProteoAnnotator reports one representative protein from each protein ambiguity group (consisting of one or more proteins) formed due to sharing the same set or subset of peptide identifications. This strategy avoids double counting of proteins with no independent evidence. We further computed the exponentially modified protein abundance index (emPAI) value as a semi-quantitative measure of protein abundance for the representative proteins using the mzidLibrary (Table S3) [38].

### SgGMMB4 methylome and codon usage

To assess whether methylation patterns differed between intact CDSs and pseudogenes, we used the ability of PacBio SMRT sequencing to detect epigenetic modifications, including (for example) 6mA, 4mC or 5mC, by comparing the sequencing profiles (specifically comparing interpulse duration) between native DNA and PCR-amplified DNA [39]; 24 869/29 832 (83.4 %) of 5-GATC-3 motifs in the SgGMMB4 chromosome are predicted to be 6-adenine methylated. No other epigenetic modifications or underlying motifs were detected. CDSs display a significantly higher frequency of methylation (4.68 per gene) than pseudogenes (2.54 per pseudogene; Kruskal–Wallis chi-squared=382.61, df=2, *P*<2.2e-16). The mean length of the CDSs is greater than that of the pseudogenes (714 bp vs 415 bp), and although the CDSs and pseudogenes did not differ significantly in their underlying mean GC content (CDSs=55.48 %, pseudogenes=55.71 %), there are therefore fewer 5-GATC-3 sites within pseudogenes (mean=three per pseudogene) than in coding sequences (mean=5.6 per CDS; Kruskal–Wallis chi-squared=428.82, df=3, *P*<2.2e-16; Table S4). There is an increased frequency of both GAT (increase of 2.7 codons per 1000) and ATC (increase of 2.2 codons per 1000) codons in CDSs vs pseudogenes (Fig. S1).

### Comparative genomics with other *Sodalis* isolates

**Fig. 4.**
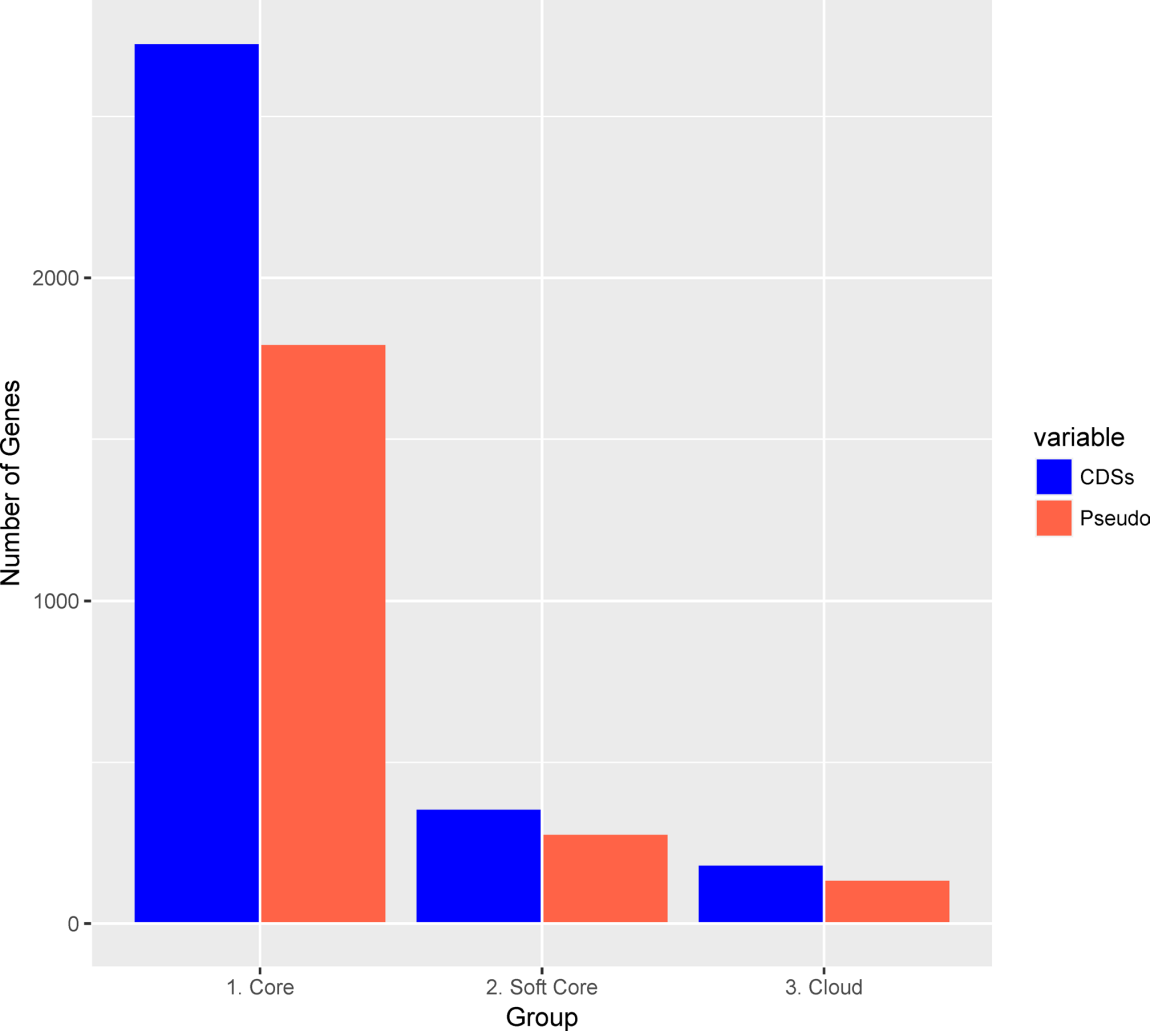
Pan-genome analysis of CDSs (blue) and pseudogenes (red) from the genomes of six *S. glossinidius* isolates derived from four different tsetse species.Plot representsthe number of CDSs/pseudogenes in the ROARY-derived pan-genome. Core, 7 genomes; soft core, 2–6 genomes; cloud, 1 genome.

Comparing single-nucleotide polymorphism (SNP) rates and pseudogene carriage between multiple genomes of *Sodalis* isolated from different tsetse hosts could reveal whether pseudogenes are being deleted at different rates, or if there is relaxed selective pressure acting on pseudogenes. ROARY pan-genome analysis of the six Illumina sequenced isolates compared to our PacBio-sequenced annotation assigned 3183 CDS and 2301 pseudogenes to either the core genome (all seven genomes), the soft core (two to six genomes) or the cloud (one genome). ROARY suggests that there are 2729 core CDSs, 358 soft-core CDSs and 184 cloud CDSs, while 1796 pseudogenes were assigned to the core genome, which represents ~40 % of the overall core genome, despite the phylogenetic distance between hosts, and there are an additional 280 soft core and 137 cloud pseudogenes (Fig. 4). Low SNP rates and stable pseudogene carriage, indicated by high numbers of pseudogenes contributing to the *Sodalis* core genome, confirms the suggestion of a recent association of *Sodalis* with the tsetse hosts, and that *Sodalis* developed an association prior to tsetse diversification. This may also imply that either pseudogenes may not be under relaxed selective pressure, due to the low rate of SNP accumulation, or that there has not been sufficient time since the association for SNPs to accumulate. Core SNPs are not targeted towards pseudogenes: core SNP using SNIPPY suggests that there are 474 core SNP loci in pseudogenes and 814 in intact CDSs, and that there are 540 intergenic core SNPs. Enriched functions of core pseudogenes are transport (GO:0005215; 144 pseudogenes) and oxidoreductase activity (GO:0016491; 92 pseudogenes). Pseudogenes that are unique to SgGMMB4 are enriched for transposase (GO:0004803; insertion sequences and phage activity).

Functional corrections of pseudogenes could be important for the ecological contribution or evolutionary trajectory of pseudogenes. To test for RNA-based (functional) corrections of genomic SNPs at the transcriptional level, a SNIPPY analysis of the RNA-seq data was performed. A single base insertion in a C(8) homopolymeric tract corrects a frameshift in a putative proline/betaine transporter (base position 869745, SGGMMB4_01173–01174/SG0498). Neither of the proP2/3 ORFs have been annotated as pseudogenes in this analysis, although no peptide has been detected for either ORF. A second insertion was found to correct for a frameshift in a putative transposase [C(9)>C(10); SGGMMB4_02124/SG0946]. Additionally, but not affecting pseudogenes, a single base insertion (G>GG) was found upstream of the annotation for the CDS SlyA_2, which is a transcriptional regulator (SGGMMB4_03264/SG1443). The SNP putatively either alters the region immediately upstream of the 5′-end or extends and alters the first 22 amino acids if reannotating using an alternative start codon. A blastp search of the sequence suggests that the current annotation for SlyA_2 is very similar to *SlyA*/*MarR* orthologues in other endosymbionts (query coverage ≥98 %; e≤4e75). Each of these SNPs was only identified in the RNA-seq data and not in the Illumina or PacBio DNA sequencing data.

## Discussion

It seems logical to consider pseudogenes as potentially maintaining a function until their association with transcriptional processes has been silenced. This is particularly pertinent in the case of secondary (S) symbionts with high proportions of pseudogenes, like *Sodalis *, which are presumed to be evolving towards an obligate association with their host. In secondary symbiosis, the current opinion is that degeneration can largely be attributed to small, vertically transmitted populations with little diversity reducing the ability of the organism to purge deleterious mutations (i.e. leading to the generation and persistence of pseudogenes) [2, 40, 41].

### Pseudogenes can harbour residual sense and antisense transcription

We have shown that bacterial pseudogenes can be both actively transcribed and dynamically regulated during growth. Expressed genes hypothesized to be under negative selection (i.e. annotations that were significantly more likely to be present in pseudogenes than CDSs) were likely to have functions related to transmembrane transport [particularly major facilitator superfamily (MFS) transporters], glycerol metabolism and transposition. MFS pseudogenes permeate genome annotations: a search for ‘MFS pseudogene’ in Ensembl bacteria shows 1984 genes at the time of writing, across many genera, including *Escherichia *, *Fransicella* and *Pseudomonas* [42]. Genes linked to substrate transport (such as MFS transporters) and metabolism are commonly found to be degraded in insect symbiont genomes, which is linked to their restricted diets [43]. Because positive selection is intrinsically linked to substrate bioavailability, as environmental conditions change (such as a lack of nutrients or the provision of a processed metabolite directly to the symbiont), relaxed selection leads to the accumulation of deleterious mutations and pseudogenization of redundant genes. Nevertheless, residual expression from these may be indicative of a recent switch in environment, where mutation at the genomic level could outpace overarching transcriptional control, so that transcription may be positively selected for due to links to other, maintained, mechanisms.

We have highlighted the related flagella and type III secretion/symbiosis regions as examples of regions undergoing clear patterns of pseudogenization (i.e. pseudogenes are clustered with few exceptions, e.g. FlgMN; Fig. 2). It is clear that coding genes associated with motility and T3SS apparatus were more likely to be differentially expressed than pseudogenes, and thus the canonical definition of pseudogenes may hold true in these cases. The FlgMN operon remains intact, despite residing within the degrading flagellum region 2 and is differentially expressed in line with flagellum region 1 (Supplementary Data 2). Additionally, the only flagellum/T3SS-related pseudogene exhibiting differential expression was flgA_2, which is the only other gene in the FlgMN operon, suggesting that pseudogene differential expression could be a factor of co-regulation of intact genes, or as a result of polycistronic regulation across operons. One of the most differentially expressed pseudogenes, without a hypothetical annotation, is trehalase, an enzyme involved in the breakdown of trehalose – a sugar commonly found in insect haemolymph [44]. Amongst a number of hypotheses as to the differential expression of trehelase in cell-free culture (which lacks trehalose as a constituent) are implications of residual global control of metabolic processes beyond single sugars. Given the importance of this sugar in insect systems, it would be interesting to further test this pseudogene for residual function.

### Pseudogenes are difficult to define

By using stranded RNA sequencing, and comparing transcription between *Sodalis* CDS and pseudogenes, we have shown that intact CDSs show significantly greater mean levels of transcription than pseudogenes, but there remains a proportion of CDSs with little or no expression – any of which could be non-functional and misannotated. It should be noted that this experiment relied on cell-free culture; CDSs may therefore not be expressed due to their functional redundancy in cell-free culture and follow-up experiments may be required in further media types, or in insect-cell co-culture, to fully ascertain the *Sodalis* transcriptional repertoire. Similarly, however, there remain pseudogenes with residual activity, going against the classical definition of a pseudogene, and it is clear, therefore, that problems remain with the identification and annotation of pseudogenes. We and others have identified novel genes, including genes potentially important in regulating flagellum and/or type III secretion machinery (*HilA*) or in quorum sensing (*SlyA*/*MarR*) [45]. Simply defining pseudogenes using any individual genomic assay is difficult: ORFs may be shortened by frameshift mutations, yet may retain functional domains and appropriate transcriptional architecture. Coding sequences are generally characterized following a set of canonical rules of gene structure: the presence of an ORF, a promoter and RBS, a methionine (or, occasionally, alternative) start codon and a stop codon. Similarly, pseudogenes were predicted wherever such rules break down: in the case of *S. glossinidius *, pseudogenes were predicted where <50 % of the functional homologue remained intact. Although studies at the single cell level in *Escherichia coli* [46], and in some conditions at the population level in *Clostridium* [47], suggest that levels of mRNA and protein can remain uncorrelated and be regulated independently of one another, our data suggest that a balance may exist between mRNA transcript and protein abundance, as a semi-quantitative measure of peptide abundance correlates with sense expression. It is likely that each tier of control (i.e. at the DNA, transcription and translation levels) may act on another – for instance, sRNA may impact on mRNA levels, or protein interactions may regulate transcription. A range of bacterial transcriptional processes remain to be fully characterized, including 5′-UTRs, alternative promoters or alternative transcriptional start and stop sites, and further experiments using techniques such as terminal endonuclease linked RNA-seq, which has been employed in similar experiments in *Salmonella enterica* serovar Typhimurium [48], would shed further light on the transcriptional landscape of this bacterium. It is important to stress that, in this study, we have defined pseudogenes specifically where peptides have *not* been detected; given the difficulty of detecting membrane proteins through proteomics analysis [49], further work may well reveal the presence of hitherto undiscovered peptides and thus that some of these differentially expressed pseudogenes may be misannotated, and it may further explain the presence of residual transcription.

### Transcriptional and post-transcriptional pseudogene control mechanisms remain to be ascertained

Given its dual role in mismatch repair and the regulation of gene expression, Dam-mediated methylation of 5-GATC-3 motifs in bacterial genomes represents a potentially important factor to investigate. While PacBio sequencing allowed for the examination of methylation status by comparing modified to unmodified DNA, the potential role methylation might play in pseudogene control remains difficult to ascertain: pseudogenes displayed a significantly decreased rate of 6mA methylation, when compared to CDSs, probably due to the tendency for pseudogenes to have fewer 5-GATC-3 methylation motifs at the genetic level (because pseudogenes are smaller than CDSs). Dam-mediated methylation is predicted to post-transcriptionally regulate gene expression by altering the affinity of proteins for DNA, such as at the origin of replication (oriC) [50]. In *S. enterica* serovar Typhimurium, adenine methylation has been implicated in regulating quorum sensing-derived virulence factors and so Dam inhibitors or Dam-silenced pathogens have been studied for their antimicrobial or vaccine potential, respectively [51]. Adenine methylation has also been implicated in protecting symbionts from heat stress [52].

### Pseudogene abundance is stable between *Sodalis* genomes

Given that we expect *Sodalis* to be routinely undergoing population bottlenecks through vertical transmission of their host, we could expect genetic drift to be acting on genes under little selective pressure, increasing SNP and/or pseudogenization rates, or even driving their deletion. As accessory genomes diverge prior to SNPs arising in the core genome [53], examining the *Sodalis* pan-genome derived from *S. glossinidius* species from multiple tsetse hosts enabled us to examine pseudogene stability.

The high number of pseudogenes in the *Sodalis* core genome suggests that pseudogenes are stable across *Sodalis* strains infecting different tsetse species – in line with the suggestion that *Sodalis* shares an evolutionarily recent association with its tsetse host due to a lack of concordant phylogeny, in contrast to that of *Wigglesworthia *, its primary symbiont [54]. Lawrence *et al*. suggested that intracellular lifestyle promotes protection from bacteriophages and insertion sequences, reducing recombination and promoting pseudogene persistence [55]. *S. glossinidius* GMBB4 has 44 chromosomal prophage elements, and an active circular phage (pSG3). Furthermore, sexual transmission of *Sodalis* from father to mother prior to vertical transmission has been reported, effectively increasing diversity and rates of recombination [56]. Assessing *Sodalis* genetic variability across wild populations of tsetse, rather than laboratory-reared tsetse, will be essential in understanding pseudogene persistence. Similarly, further research into the expression levels of both coding and pseudogene orthologues may reveal further insights into transcriptional co-regulation – and gene redundancy linked to reductive evolution [57]. Kuo and Ochman have previously suggested that *Salmonella* pseudogenes may lack sufficient negative pressure for deletion [12]. In models of cyanobacterial genomes, increased resource levels and decreased mortality have been suggested to select for slower reproduction and streamlined genomes [58]. Experimental evolution experiments in *Methylobacterium* have shown that accessory gene deletion confers a direct fitness benefit under selective environments, rather than the associated benefit of the reduced fitness costs of maintaining a shorter genome in its own right [59], and similar experiments would be interesting to perform in this system.

Defining which associations constitute significant functions, on account of which positive pressure ensures that they persist, is difficult: associations with promoters, transcription factors or *cis*/*trans*-acting transcriptional regulators could all select for pseudogene retention in the genome, and reduce the likelihood of full deletion. An increasing number of bacterial small RNAs have been identified through transcriptomic analyses, including in *Streptococcus* [60] and *Borrelia* [61]. Pseudogene-derived antisense RNA may be involved in the complex interactions between genome, transcriptome and proteome: sRNAs have been implicated in gene regulation of multiple target genes through processes such as translational inhibition and activation, or transcript stability [62]. *Hfq*, a crucial chaperone involved in bacterial sRNA processing, is maintained in *Sodalis* (SGGMMB4_00878). Therefore, further studies into the roles of *Sodalis* sRNAs – including pseudogene-derived sRNAs and the role of *Hfq* or other chaperones – will be critical for full understanding of the complexity of gene regulation in this degrading bacterial genome. This is in line with previous work using tiling arrays in *Mycoplasma pneumonia*, wherein frequent antisense and non-coding transcripts were identified in a degrading bacterial genome [63]. Pseudogene-derived transcripts (such as antisense small RNAs derived from pseudogenes) could act as regulators for orthologues elsewhere in the genome [64] and such an association may reduce the selective pressure towards their deletion. As this study relied on RNA shearing-based library preparation, it would be interesting to follow it up with full-length third-generation RNA sequencing [i.e. using PacBio cDNA (Isoseq) or direct RNA sequencing using Oxford Nanopore technologies] to elucidate the sequences of full-length mRNA, including primary, polycistronic transcripts, which would further enhance our knowledge as to how pseudogenes continue to contribute to overall transcription and its control despite ongoing genomic degradation.

## Conclusions

The primary goal of this study was to establish whether bacterial pseudogenes remain active despite genomic degradation, using *Sodalis* as a model given the number of putatively inactivated, functionless genes persisting in its genome. We sought to combine DNA sequencing, stranded RNA sequencing and proteomic analysis to fully describe the *Sodalis* transcriptional and translational landscape with a view to better understand the evolution and functional control of bacterial pseudogenes and the process of endosymbiont genome degradation.

We have revealed that whilst transitioning from a free-living to a symbiotic status, *Sodalis* pseudogenes are often transcribed, but at a significantly lower level than intact CDSs. Some pseudogenes even remain under active transcriptional control, exhibiting differential expression throughout growth, but proteomic analysis suggests that they ultimately do not contribute to the protein content of the cell. The lack of some expression from intact CDSs and pseudogenes underpins the difficulty in pseudogene identification – especially in cell-free culture, where the correct conditions for their expression may be lacking. The fact that a combination of sense and antisense transcription of pseudogenes persists implies a role of pseudogene transcription in control mechanisms, e.g. cis/*trans* small RNA transcriptional control, and could even be playing a role in wide-reaching mechanisms such as host–symbiont interaction, or symbiont–symbiont interaction. The persistence of pseudogenes in the *Sodalis* pangenome implies that the maintenance of the function of degraded genes may outweigh any deleterious effects, or that there is a mechanism through which such deleterious effects are mitigated. Given the proximity of *Sodalis* to medically important parasites and other bacteria within the tsetse host, further study on these mechanisms is of interest for the identification of novel therapeutic interventions.

## Methods

### DNA sequencing

For PacBio sequencing, *S. glossinidius* strain GMMB4 (SgGMMB4) was isolated from *Glossina morsitans morsitans* (Westwood) from the Langford-derived long-term colony maintained at the University of Edinburgh in 2005. Six further *S. glossinidius* isolates were cultured from laboratory-based tsetse for Illumina sequencing: GP1 and GPP4 were isolated from *Glossina palpalis palpalis*; GAA from *G. austeni*; GF4 from *G. fuscipes*; and GM1 and GMM4 from *G. morsitans* as previously described [65].

Bacteria were recovered from −80 °C storage by incubation at 25 °C on Columbia agar plates supplemented with 10 % defibrinated horse blood (TCS Biosciences) in microaerophilic conditions (~5–12 % CO_2_; CampyGen, Oxoid, UK). An individual colony was picked and grown to late stationary phase in cell-free culture medium at 25 °C in Schneider’s insect medium (Sigma, UK) supplemented with 10 % foetal calf serum (Life Technologies, UK). High-molecular-weight whole=genomic DNA (gDNA) was extracted from the subsequent bacterial pellet using the Zymo Research Universal gDNA extraction kit (SgGMMB4) or the Qiagen DNeasy kit (other isolates) according to the manufacturer’s instructions.

SgGMMB4 gDNA was sequenced on the Pacific Biosciences RS-II instrument (PacBio) at the Centre for Genomic Research at the University of Liverpool on a single SMRT cell using P6-C4 chemistry with no prior size selection. Reads were assembled and contigs polished using HGAP.3, resulting in a polished assembly consisting of a single chromosomal contig and nine further contigs. Comparison of the sequence to the available reference by MUMMER [66] and ACT [67] suggested that two contigs were a result of chimeras derived from pSG2. A further five contigs were repetitive phage-derived sequences. The chromosome was subsequently manually edited to begin at the start of the dnaA gene. The putative protein-coding, ncRNA and tRNA gene sequences were annotated using PROKKA (v. 1.10) [68]. Pseudogenes in this study were initially conservatively annotated where the PROKKA-defined ORFs overlapped with the Belda-annotated pseudogenes or predicted to be pseudogenes by PROKKA [68]. In the latter case, PROKKA predicted pseudogenes based on identical annotations in sequential ORFs (except for hypothetical protein annotations). Sequences matching possible riboswitch domains were predicted using the Denison Riboswitch Detector online tool [69]. Additionally, the two available annotations for SgGMM4 [14, 23] were transferred to the PacBio SgGMMB4 scaffold using the RATT software package [33], for comparison and pseudogene prediction (Supplementary Data 4). An additional *S. glossinidius* sample (isolated from *G. palpalis*) was sequenced and assembled in the same manner using two SMRT cells using P6-C4 chemistry.

Sequencing libraries for the six further isolates of *S. glossinidius* from multiple tsetse species (GAA; GF4; GM1; GMM4; GP1; GPP4) were prepared using a TruSeq library preparation kit (according to the manufacturer’s instructions) and sequenced on a single lane of an Ilumina HiSeq (high-output run; 2×100 bp paired end reads) at the Centre for Genomic Research. The Illumina HiSeq data from the six further *S. glossinidius* isolates were initially processed using CASAVA 1.8 to produce FASTQ files. FASTQ data files were trimmed for the presence of Illumina adapter sequences using Cutadapt (v1.2.1), using the –O 3 option [70]. The reads were further trimmed using Sickle (v. 1.200) (https://github.com/najoshi/sickle) with a minimum window quality score of 20. Data were assembled *de novo* with SPADES using default parameters and annotated using PROKKA as previously described. PROKKA-derived GFF annotations were processed through the ROARY pan-genome package to ascertain core and accessory genome coverage [71]. Reads were mapped to the SgGMMB4 PacBio reference, and core SNP phylogenies derived using the SNIPPY package (https://github.com/tseemann/snippy).

### Methylome sequencing

In addition to gDNA sequencing and assembly, the PacBio RSII instrument can detect epigenetic modification either *in silico* or by comparing native DNA to a PCR control. To that end, a whole-genome-amplification- (WGA) control was generated from SgGMMB4 as follows: 1 µg gDNA was split into three equal reactions and whole-genome amplified using the Qiagen Repli-g Turbo kit according to the manufacturer’s instructions. These were then pooled and cleaned using a 2 : 1 ratio of homemade SPRI bead cleanup system analogous to Ampure XP beads [72]. The WGA control was sequenced using one SMRT cell in the same way as described previously. Comparison to the native DNA was performed using the Motifs and Modifications module within the SMRT Analysis Server with a mapping quality cutoff set at QV70 and the subsequent modifications and motifs file was filtered for those with a quality ≥Q50 (*P*<0.0001).

### RNA sequencing

Individual 10 ml cell-free liquid cultures, as described above, were set up in quintuplets for 17 time-points at 6-hourly intervals. At each time point, the contents of the culture flasks were transferred to 15 ml Falcon tubes, an optical density (600 nm) measurement was taken, and then the tubes were centrifuged for 10 min at 10 °C. The bacterial pellet was immediately resuspended in 1 ml Trizol reagent (Life Technologies) and total RNA was extracted using Zymo Research DirectZol columns. RNA cleanups were performed using a 2 : 1 ratio of SPRI beads as described previously. Three timepoints representing the early log (12 h); late log (72 h) and late stationary phases (108 h) according to the OD measurements were selected and DNase I treated using a Life Technologies DNAfree Turbo kit according to the manufacturer’s instructions (data not shown).

Ribosomal RNA (rRNA) was depleted using a Ribo-Zero bacterial (low-input) rRNA Removal kit (Epicentre), and individually barcoded, strand-specific Illumina cDNA libraries were prepared using a NEBNext Ultra RNA Library Prep kit for Illumina. Sequence data were generated using one Illumina MiSeq run with v2 chemistry, generating 250 bp paired-end reads. All RNA and cDNA cleanups were performed using SPRI beads as described previously. All raw Illumina sequence Fastq files were trimmed for the presence of adapter sequences using Cutadapt v. 1.2 using option −O 3 [70] and quality-trimmed using Sickle v. 1.200 [73] with a minimum window quality score of 20. Any reads shorter than 10 bp after trimming were removed. Quality scores for all sequences were assessed using FASTQC v0.9.2 (www.bioinformatics.babraham.ac.uk/projects/fastqc). RNA-seq reads were aligned to the PacBio RSII-generated SgGMMB4 scaffold using Bowtie 2 v. 2.1.0[74]. The resulting SAM files were converted to BAM and sorted using samtools v. 0.1.18-r580s [75]. For transcript-based annotation, reads were counted against the PROKKA/PacBio annotation using HTSeq version 0.5.3p9 using the --stranded option, to count in both the sense and antisense directions, and using the intersection-nonempty mode [76]. EdgeR analysis was implemented using the DEGUST web package (http://degust.erc.monash.edu), which outputs counts per million, logFC and differential expression statistics (Supplementary Data 1). EdgeR counts per million were transformed to TPM according to Wagner *et al*. [77]. Further statistical analysis and figure plotting were implemented using R version 3.32. Operon structure was predicted from the RNA-seq data using Rockhopper with the default settings [78]. The combined RNAseq sequencing reads were fed through the SNIPPY pipeline (as previously described) to identify potentially correcting SNPs. Gene enrichment analyses were performed in blast2GO 5, using Interpro GO IDs (Supplementary Data 3).

### Proteomics

Liquid cultures were grown as previously described for early-log, mid-log and late-stationary growth phase *S. glossinidius* GMMB4. Phosphate-buffered saline (PBS)-washed pellets were suspended in 250 µl of 25 mM ammonium bicarbonate and sonicated using a Sonics Vibra Cell (Sonics and Materials, Inc., Newton, USA) and 630–0422 probe (250 µl to 10 ml) for a total of 120 joules. The sample was then analysed for protein content, 50 µg was added to 0.05 % RapiGest (Waters, Manchester, UK) in 25 mM ammonium bicarbonate and shaken at 550 r.p.m. for 10 min at 80 °C. The sample was then reduced (addition of 10 µl of 60 mM DTT and incubation at 60 °C for 10 min) and alkylated (addition of 10 µl of 180 mM iodoacetamide and incubation at room temperature for 30 min in the dark). Trypsin (proteomics grade; Promega UK, Southampton, UK) was reconstituted in 50 mM acetic acid to a concentration of 0.2 µg µl^−^^1^ and 10 µl was added to the sample followed by overnight incubation at 37 °C. The digestion was terminated and RapiGest was removed by acidification (1 µl of TFA and incubation at 37 °C for 45 min) and centrifugation (15 000 ***g*** for 15 min). To check for complete digestion, each sample was analysed pre- and post-acidification by SDS-PAGE.

For liquid chromatography/tandem mass spectrometry (LC-MS/MS) analysis, a 2 µl (1 µg) injection was analysed using an Ultimate 3000 RSLC nano system (Thermo Scientific, Hemel Hempstead, UK) coupled to a QExactiveHF mass spectrometer (Thermo Scientific). The sample was loaded onto the trapping column (PepMap100, C18, 300 µm×5 mm, Thermo Scientific) using partial loop injection for 7 min at a flow rate of 4 µl min^−1^ with 0.1 % (v/v) FA. The sample was resolved on the analytical column (Easy-Spray C18 75 µm×500 mm 2 µm column) using a gradient of 97 % A (0.1 % formic acid), 3 % B (99.9 % ACN 0.1 % formic acid) to 70 % A, 30 % B over 120 min at a flow rate of 300 nl min^−1^. The data-dependent program used for data acquisition consisted of a 60 000 resolution full-scan mass spectrometry (MS) scan (AGC set to 3e6 ions with a maximum fill time of 100 ms). The 18 most abundant peaks were selected for tandem MS (MS/MS) using a 30 000 resolution scan (AGC set to 1e5 ions with a maximum fill time of 45 ms) with an ion selection window of 1.2 *m*/*z* and a normalized collision energy of 28. To avoid repeated selection of peptides for MSMS the program used a 30s dynamic exclusion window.

The protein identification for the MS/MS dataset was performed using an open-source software tool – ProteoAnnotator [79]. ProteoAnnotator provides an automated pipeline for various interconnected computational steps required for inferring statistically robust identifications. The tool produces a variety of output files compliant with the data standards developed by Proteomics Standard Initiative [80]. Mass spectra in form of a Mascot generic format (MGF) file were provided as input to the tool, along with the search criteria and protein database. The search parameters for the MS/MS dataset were fixed modification of carbamidomethylation of cysteine and variable modification of oxidation of methionine. A single missed trypsin cleavage was allowed. The product tolerance was set as ±0.5 Da and the precursor tolerance was set as 10 p.p.m. The protein search database comprised the gene model predicted by PROKKA, as previously described, plus a six-frame translation of the SgGMMB4 genome. Six-frame translated sequences with a length of <8 were excluded from the search database. Decoy sequences were added to the database with a true : decoy ratio of 1 : 1 to create a final protein database for performing the MS/MS search. For the post-processing of results, we applied a threshold of 5 % for both the peptide level and the protein group level FDRs, as described in Ghali *et al*. (2004) [79].

## Data bibliography

1. Centre for Genomic Research. Genomes of *Sodalis glossinidius* isolates. NCBI Nucleotide Archive Project PRJEB9474 (ENA-SUBMISSION: ERA441631) (2016).

2. Centre for Genomic Research. Stranded RNA sequencing of *Sodalis glossinidius* - a bacterial endosymbiont of the Tsetse fly. NCBI Nucleotide Archive Project PRJEB20150 (2017).

3. Centre for Proteome Research. Proteome of *Sodalis glossinidius* strain GMMB4. Proteomexchange ID: PXD007068 (2016).

## Supplementary Data

Supplementary material 1

Supplementary material 2
