# Supplementary material for: Large-scale and significant expression from pseudogenes in Sodalis glossinidius – a facultative bacterial endosymbiont
Source: Microb Genom. 2020 Jan 10;6(1):e000285. doi: 10.1099/mgen.0.000285 (PMC7067036; doi:10.1099/mgen.0.000285)

Supplementary Data 5: “Pseudogenised” genes with residual active translation

Seven pseudogenisation events that split ancestral orthologues into two-or-more ORFs yield a protein product from one of the resultant ORFs. A further 27 single-ORF PROKKA predictions that overlap Belda-predicted pseudogenes were identified to produce a corresponding unique peptide, detailed in Supplementary Table S2.

hrpB encodes an ATP-dependent DExH-box RNA helicase that may melt short RNA duplexes, rearrange RNA secondary structure, and may play a role in in protein-RNA interaction (71). SgGMMB4 has six RNA helicases, however hrpB is the only pseudogenised example. hrpB has been pseudogenised by an internal stop-codon and a subsequent frame-shift, effectively splitting the gene into three ORFs. Both of the first two hrpB ORFs exhibit detectable mRNA, no antisense transcription, and the first ORF exhibits detectable protein despite later pseudogenisation. BLASTP of the first hrpB ORF shows ≥98% similarity to partial helicases in other enteric microbial species.

SgGMMB4_01425 (ps_SGL0337) is a hypothetical protein pseudogenised into four ORFs (five by Belda *et al.*) by frameshift mutations, all of which exhibit significant sense transcription. The first of these ORFs is translated to detectable protein. BLASTP shows 85% identity (60-70% coverage) of an aldolase gene, which are a group of genes involved in glycolysis and gluconeogenesis. Proteomics suggests the presence of at least seven more functional aldolase genes in the SgGMMB4 genome.

SgGMMB4_01979 - SgGMMB4_01981 (SG0895; GalK) is a galactokinase gene predicted to have three consecutive ORFs by PROKKA. All three ORFs are actively transcribed. The first ORF is identical to the Toh and Belda annotations, however the subsequent two were not annotated. Interestingly, the second ORF (SgGMMB4_01980) yields a detectable peptide by proteomics. There are no other galactokinase genes predicted in the genome. BLASTP suggests ≥70% identity and ≥96% coverage to similar galactokinase genes in other Enterobacteriaceae species (data not shown).

SgGMMB4_02516 - SgGMMB4_02517 (ps_SGL0606) is a yeeA inner membrane protein pseudogenised by a frameshift mutation. Nonetheless, the frameshift results in a functional second ORF with a methionine start codon. Both ORFs are actively transcribed, however only the second ORF yields a functional protein product as detected by a unique peptide signature from proteomic analysis.

SgGMMB4_03000 (ps_SGL0739) is a SlyA transcriptional regulator, responsible for controlling numerous virulence factors via the PhoP/PhoQ two-component system in Salmonella. Indeed, SlyA also acts as a positive feedback loop, by binding to the phoPQ promoter in high Mg^{2+} conditions (72). Not annotated in the Toh *et al.* annotation and predicted to be a pseudogene in the Belda *et al.* annotation, SlyA shows active sense and antisense transcription and translation. SgGMMB4 has a second SlyA (SG1443 / SgGMMB4_03264) that is actively transcribed and translated, and is not predicted to be a pseudogene by Belda *et al*.

SgGMMB4_04819 - SgGMMB4_04822 (speC; ps_SGL1267c) encode an ornithine decarboxylase gene containing two or three frameshifts (depending upon the annotation) and an internal stop-codon between ORFs two and three. Sense transcription, and a unique peptide, could only be detected for the first ORF (SgGMMB4_04822, negative strand), but none of the other three (as predicted by PROKKA), although antisense transcription was present for the final two ORFs.

SgGMMB4_04854 is the first of two ORFs represented by ps_SGL1277 that contains a premature stop codon. Proteomics detects a putative exported protein from the ORF, for which sense and antisense transcription and a viable peptide product can be detected. No protein is detected for SgGMMB4_04855 despite showing similar levels of expression on both strands.

Figure S1: Codon Usage.

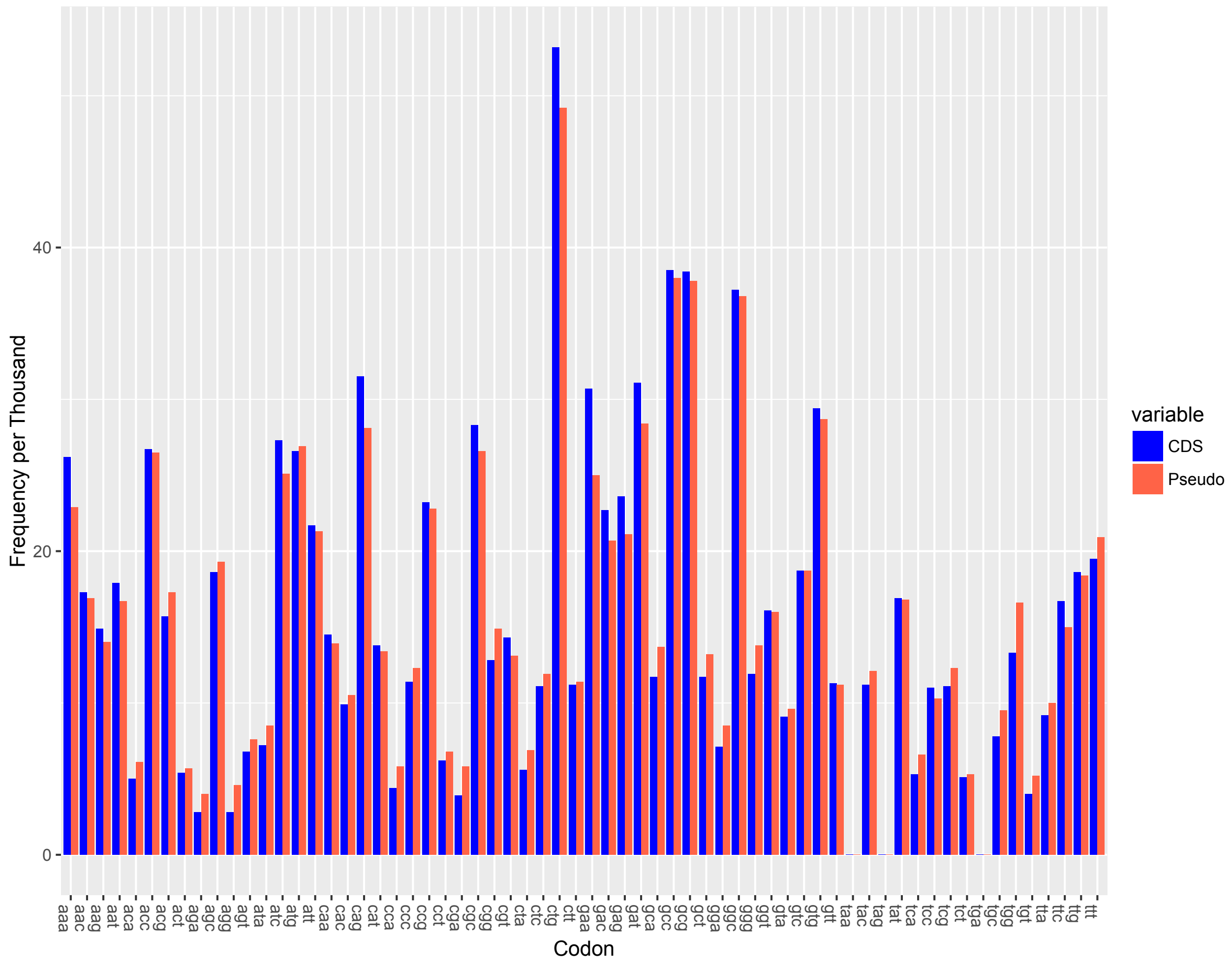

Supplement: Supplementary material 1 [file mgen-6-285-s001.pdf]
